# Shaping applied epidemiology workforce training to strengthen emergency response: a global survey of applied epidemiologists, 2019–2020

**DOI:** 10.1186/s12960-021-00603-1

**Published:** 2021-04-29

**Authors:** Amy Elizabeth Parry, Martyn D. Kirk, David N. Durrheim, Babatunde Olowokure, Samantha M. Colquhoun, Tambri Housen

**Affiliations:** 1 National Centre for Epidemiology and Population Health, Research School of Population Health, The Australian National University, Building 62 Mills Road, Acton, Australian Capital Territory, Australia; 2 University of Newcastle, Newcastle, New South Wales Australia; 3 World Health Organization, Health Emergency Information and Risk Assessment, Geneva, Switzerland

**Keywords:** Epidemiology, Public health practice, Health workforce, Emergency, Education, Emergencies

## Abstract

**Background:**

Rapid and effective emergency response to address health security relies on a competent and suitably trained local and international workforce. The COVID-19 pandemic has highlighted that the health security workforce needs to be well equipped to tackle current and future challenges. In this study, we explored whether training in applied epidemiology was meeting the current needs of the applied epidemiology workforce.

**Method:**

We conducted a cross-sectional online survey that was available in English and French. We used purposive and snowballing sampling techniques to identify potential survey respondents. An online social media advertisement campaign was used to disseminate a REDCap survey link between October 2019 and February 2020 through field epidemiology networks. Survey questions included demographic details of participants, along with their technical background, level of formal education, topics studied during epidemiology training, and years of experience as an epidemiologist. We used Pearson Chi-squared (Chi^2^) to test the difference between categorical variables, and content analysis to evaluate responses to open-ended questions.

**Results:**

In total, 282 people responded to the survey. Participants had a range of formal public health and epidemiology training backgrounds. Respondents applied epidemiology experience spanned almost 30 years, across 64 countries. Overall, 74% (*n* = 210) were alumni of Field Epidemiology Training Programs (FETP). Basic outbreak and surveillance training was well reported by respondents, however training in specialised techniques related to emergency response, communication, and leadership was less common. FETP graduates reported higher levels of formal training in all survey topics.

**Conclusion:**

It is critical for the health security workforce to be well-trained and equipped with skills needed to ensure a rapid and effective response to acute public health events. Leadership, communication, interpersonal skills, and specialist training in emergency response are lacking in current training models. Our study has demonstrated that applied epidemiology workforce training must evolve to remain relevant to current and future public health challenges.

## Background

Rapid and effective emergency response to address health security challenges relies heavily on a competent and suitably trained local and international workforce [1, 2]. The COVID-19 pandemic highlighted the importance of having a well-equipped health security workforce. Future epidemics of emerging infectious disease and other acute public health emergencies will continue to occur. To respond to these events, it is essential that training programs are providing graduates with the skills they need [3].

Well-trained field and applied epidemiologists are a crucial component of the health security workforce to prepare and effectively respond to health emergencies [1, 4]. Applied epidemiologists work in government and non-government organisations to detect, investigate, manage, and control infectious diseases [1, 4, 5], and have been described as “activists” who rapidly transform findings into policy and action [6].

Responsibility for applied epidemiology workforce development varies depending on the country [7, 8]. The Field Epidemiology Training Program (FETP) model [7] is a learn-by-doing training approach [4, 8, 9], where trainees are imbedded within their national health system. FETPs aim to improve public health systems through strengthening disease surveillance for evidence-based decision-making, and enhancing capacity for outbreak prevention, detection and response [8]. This training can vary in duration from 3 months for ‘frontline’ programs through to the 2 years for ‘advanced’ programs and are largely supported by Government health departments [10]. Academic institutions provide Master of Public Health or Epidemiology Programs, offering training in basic or advanced epidemiology, and biostatistics, with some offering specialist training in disaster management and/or humanitarian crisis management [11, 12].

The Ebola outbreak in West Africa, and more recently the COVID-19 pandemic, demonstrated the fragility of national and international public health response models [2]. These public health events have exposed the broader health system and health security implications of under-investing in public health systems and training field epidemiologists [2]. There is an urgent need to review the investment in health security workforce development, to ensure countries have an appropriately skilled and confident workforce to rapidly manage and contain public health emergencies.

In 2019, we interviewed public health experts about the needs and challenges of the epidemiology emergency response workforce. Experts outlined the need for collective competence and discussed the lack of training standardisation, the need for skills in leadership, communication, and also specialist training in emergency response [13]. To understand if current models of applied epidemiology training were meeting the needs of the emergency response workforce and to identify training needs, we conducted an online survey administered to applied epidemiologists. We analysed survey data to understand the knowledge and skills obtained during education and training in applied epidemiology.

## Methods

**Fig. 1:**
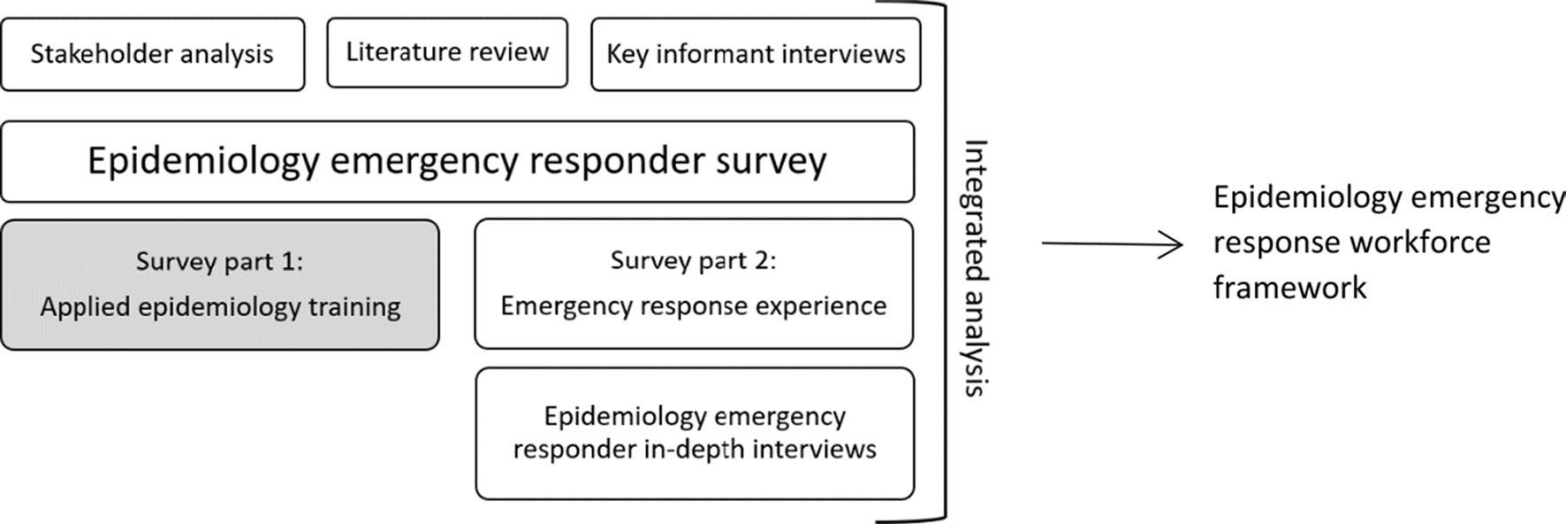
Epidemiology emergency workforce research study model [14]

As part of a larger study looking at the effectiveness of the epidemiology workforce during emergency response [14], (Fig. 1) we conducted an online cross-sectional survey to identify training needs from the perspective of applied epidemiologists. For the purposes of this study, the ‘applied epidemiology workforce’ was defined as any person working in an applied or field epidemiology role or acute public health responder role.

### Study population

The target population for this survey was the global applied epidemiology workforce. We specifically targeted FETP alumni, however the survey was open to all people who had studied epidemiology and used this training in a practical setting.

### Sampling

As there was no defined and reliable sampling frame, a combination of purposive and snowballing sampling techniques was used to identify the target population [15, 16].

Although the size of our study population for this survey was unknown due to our multiple recruitment methods, the primary means for survey distribution was the TEPHIConnect field epidemiology database, with 1700 registered active members as of September 2019. Using this as an indicative sampling frame, and the response rate from a previous unpublished survey by TEPHIConnect of 9.8%, we assessed that the final sample size of 282 in this study would to lead to estimates of a single proportion with a 95% confidence interval, with precision of ± 6%, after employing the finite population correction.

### Recruitment

We recruited survey respondents through multiple sources. A YouTube video described the purpose of the study in English; this video included the option of subtitles in English or French. This video formed the basis of a social media recruitment campaign through Twitter, LinkedIn, and our dedicated study Facebook page. We disseminated participation reminders eight times through these social media channels over a 3-month period. We partnered with the global field epidemiology training alumni network, TEPHIConnect, who ran a social media campaign mirroring our campaign. The campaign stated that any person working in an applied epidemiology role could complete the survey. Individuals self-selected based on this criterion.

Postcards advertising the survey were distributed at the 10th TEPHINET Global Scientific Conference, the Australian Communicable Diseases Control Conference 2019, and the European Scientific Conference on Applied Infectious Disease Epidemiology (ESCAIDE 2019). In addition, the study population were directly invited to participate in the survey by email via field epidemiology networks including TEPHIConnect and national FETP networks. We encouraged survey respondents to forward the survey to their personal and professional network.

### Consent

Participation in this study was voluntary; participants were directed to plain language information sheets, available online in French and English, prior to being able to access the survey. Each participant provided online consent prior to obtaining access to the survey questions. Respondents had the option to navigate the survey and remove or change answers prior to submission.

### Ethics

This survey was approved by the Australian National University Human Research Ethics Committee, ID 2019-068.

### Data collection

We used findings from key informant interviews conducted in 2019 [13, 17] and literature reviews to develop survey questions [18]. The questionnaire was reviewed by key informants and pre-tested with 11 individuals representative of the target population to test for construct and content validity.

The survey was divided into three modules: demographics, epidemiology training, and deployment experience. This paper reports findings from the first two modules. The demographics module included age, gender identity, citizenship, formal education, technical background, years of epidemiology experience, and emergency response experience. For FETP trainees and graduates, we asked about their highest FETP level achieved, based on the globally recognised categories of frontline (≤ 6 months), intermediate (9 months–1 year), and advanced (2 years) tiers [9].

The survey module on epidemiology training included five broad areas, each with a checkbox list of specific training items: surveillance (6 items), data analysis and assessment (15 items), leadership (9 items), social and communication skills (11 items), and emergency response (14 items). We termed what is commonly known as ‘soft skills’ or ‘interpersonal skills’ [19] as ‘social and communication skills’. The variation in terminology for this set of skills varied across the literature with little consensus, either too narrow in definition for this study or unclearly defined.

This module captured qualitative data via open-ended questions with free-text fields where respondents could identify further training gaps for each area, provide further comments, or list additional training received. Respondent were also asked to comment on whether the knowledge and skills gained during their training, and were appropriate and adequate for emergency response.

We developed and tested the survey in English, which was then translated into French. A native French-speaker re-checked the translation to ensure accuracy of meaning. The survey was self-administered online by respondents via REDCap (Research Electronic Data Capture) [20] between October 2019 and February 2020. No incentives were provided for participation.

### Data analysis

Survey data were analysed descriptively in Microsoft Excel (2016) and Stata15 (TX:StataCorp) [21]. Data were analysed to explore associations within and between respondents. We compared self-recounted epidemiology training between FETP and non-FETP and gender. Reported FETP levels were not sub-analysed due to low numbers of respondents from frontline and intermediate programs. We used Pearson Chi-squared (Chi^2^) to test for significant differences between categorical variables. Answers to open-ended questions were analysed using Nvivo11 [22], data familiarisation led to open-coding of text; common categories were created iteratively and merged or expanded as needed [23, 24].

We used the six World Health Organization (WHO) regions to categorise respondents from different geographical areas according to: the Regional Office for Africa (AFRO), Regional Office for the Eastern Mediterranean (EMRO), the Regional Office for Europe (EURO), Pan American Health Organization (PAHO), South-East Asia Regional Office (SEARO), and the Western Pacific Region Office (WPRO) [25].

## Results

Three hundred and thirty individuals consented to participate, of whom 282 (85%) completed the survey; 268 (95%) in English and 14 (5%) in French.

Respondents were able to select multiple options regarding how they were informed of the survey, 104 (32%) indicated Facebook, Twitter, and/or LinkedIn, similarly 105 (32%) indicated the TEPHINET/TEPHIConnect alumni network, 71 (22%) indicated they were notified through their country specific FETP network and 42 (13%) through personal or professional contact snowballing sampling.

### Demographics

Gender distribution of respondents was similar, 51% female (*n* = 144/282). The median age of respondents was 39 (range: 23–77 years) (Table 1). Gender, age distribution, and education level were similar between FETP and non-FETP respondents.

**Table 1:** Demographic characteristics of epidemiology emergency response survey respondents, 2019–2020 (*n* = 282)

Category	Variable	Number (%)
Gender		
	Female	144 (51.1%)
	Male	135 (47.9%)
	Non-conforming	0 (0%)
	Prefer not to answer	3 (1.1%)
Age (years)		
	< 20	0 (0%)
	20–29	18 (6.4%)
	30–39	140 (49.6%)
	40–49	78 (27.7%)
	50–59	37 (13.1%)
	60–69	7 (2.5%)
	70 +	2 (0.7%)
Technical background^a^		
	Epidemiology	225 (79.8%)
	Public Health	158 (56%)
	Medicine	70 (24.8%)
	Laboratory	35 (12.4%)
	Nursing	25 (8.9%)
	Data science	21 (7.4%)
	Veterinary	19 (6.7%)
	Social Science	14 (5.0%)
	Other	23 (8.2%)

^a^Multiple technical backgrounds per respondent

The majority of respondents reported a professional or technical background in epidemiology (79.8% *n* = 225/282, Table 1) with postgraduate or doctorate-level qualifications (91.5% *n* = 258/282, Table 2). When disaggregated by gender, there were twice as many male laboratory specialists (65.7% *n* = 23/35 *p* = 0.028) and significantly more female social scientists (78.5% *n* = 11/14 *p* = 0.038). There were more females who identified as epidemiologists (53% *n* = 120/225), nurses (64% *n* = 16/25), and public health specialists (53% *n* = 84/158), however, a statistically significant difference was not detected.

**Table 2:** Professional background characteristics of epidemiology emergency response survey respondents, 2019–2020 (*n* = 282)

Category	Variable	Number (%)
Epidemiology and Public heath training^a^		*n* = 282
	Master of Public Health (or similar)	92 (32.6%)
	Other relevant Masters	13 (4.6%)
	Public Health Physician training	10 (3.5%)
	PhD	18 (6.4%)
	Field Epidemiology Training Program (FETP)	210 (74.5%)
FETP		*n* = 282
	Yes	178 (63.1%)
	Trainee at time	32 (11.3%)
	No	72 (25.5%)
FETP level		*n* = 210 (including trainees)
	Advanced (2 years)	189 (90%)
	Intermediate (9 months – 1 year)	7 (3.4%)
	Frontline (< 6 months)	7 (3.4%)
	Unanswered	7 (3.4%)
Years since FETP graduation		*n* = 210
	10 or less	157 (74.8%)
	10 +	37 (17.6%)
	Unanswered	16 (7.6%)
Epidemiology experience		*n* = 282
	< 1 years	12 (4.3%)
	1– < 2 years	23 (8.2%)
	2– < 5 years	77 (27.3%)
	5– < 8 years	64 (22.7%)
	8– < 12 years	42 (14.9%)
	12+ years	59 (20.9%)
	Not applicable	4 (1.4%)
	Unanswered	1 (0.4%)

^a^Multiple training types per respondent

**Fig. 2:**
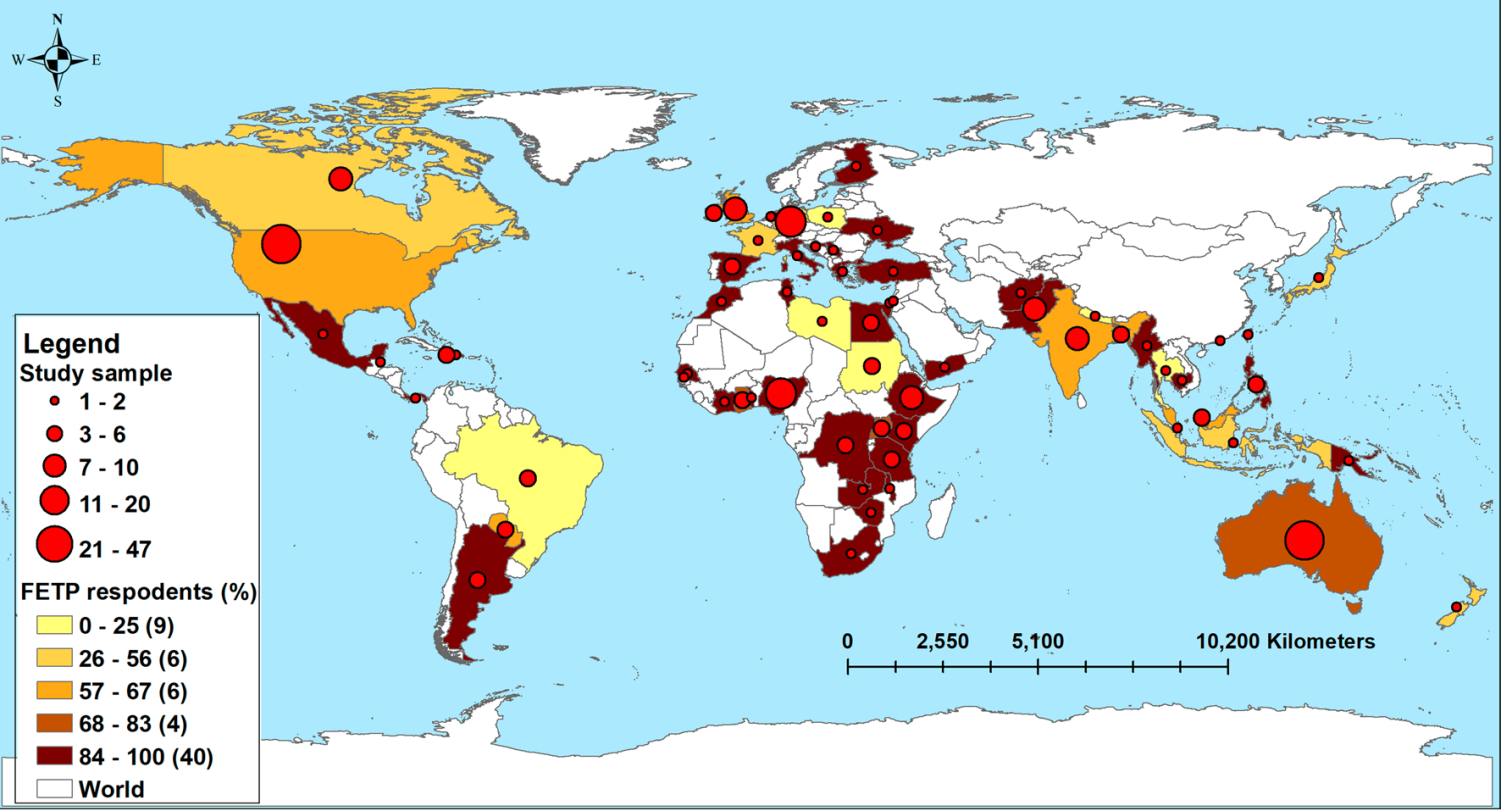
Distribution of epidemiology emergency response survey respondents by country and training type, 2019–2020 (*n* = 282)

Respondents came from 64 countries, with the highest proportion from the United States of America (16.7% *n* = 47/282), Australia (13.8% *n* = 39/282), and Nigeria (7.1% *n* = 20/282). When stratified by WHO regions, the highest response was from the America’s (PAHO) (Fig. 2). Respondents in AFRO and SEARO were more likely to be male (76% *n* = 51/67, 85% *n* = 23/27, respectively), with a higher proportion of female respondents in PAHO and WPRO (69% *n* = 53/76, 71% *n* = 41/57, respectively). Gender distribution within EMRO and EURO respondents was similar. Age distribution across all regions was similar and FETP respondents made up between 65% and 89% of respondents (Fig. 2).

Respondents reported a variety of formal public health and epidemiology training, including, FETP, postgraduate master’s programs, doctorate programs, physician public health training, and short courses in specified public health and epidemiology topics (Table 2). The majority of respondents (74%, *n* = 210/282) indicated they were graduates or trainees of an FETP, 90% (*n* = 189/210) of whom reported to have studied the advanced program (Table 2). Respondents had predominately (74.8%,* n* = 157/210) graduated from their FETP within the past 10 years (Table 2), with the earliest graduation being 1992. Professional epidemiology experience varied with 40% (*n* = 112/282) of respondents reporting less than 5 years, and 20% (*n* = 59/282) reporting more than 12 years of experience (Table 2).

## Epidemiology training

### Outbreak and surveillance

Six outbreak and surveillance categories were listed in the survey (Table 3a). Training in general outbreak and surveillance was commonly reported by the respondents, however less commonly reported was targeted training in emergency response surveillance, community-based surveillance or syndromic surveillance (Table 3a). When comparing the respondents who had completed an FETP with those who had not participated in an FETP, FETP graduates reported higher levels of formal training in all surveillance and outbreak investigation categories (Table 3a).

**Table 3:** Reported epidemiology training, comparison between FETP and non-FETP epidemiology emergency response survey respondents, 2019–2020 (*n* = 282)

Section	Category	Topic	Total *n* = 282	Total %	Non-FETP *n* = 72	Non-FETP%	FETP *n* = 178	FETP%
a	Training in outbreak and surveillance							
		Basic principles of surveillance	262	92.9	58	80.6	173	97.2
		Syndromic surveillance	165	58.5	23	31.9	118	66.3
		Community-based surveillance	157	55.7	34	47.2	104	58.4
		Emergency response surveillance	168	59.6	29	40.3	119	66.8
		Outbreak investigation steps	250	88.7	51	70.8	168	94.4
		Outbreak investigation methods	238	84.4	47	65.3	160	89.9
b	Training in analysis and epidemiology methods							
		Rapid survey	162	57.4	35	48.6	107	60.1
		Mortality survey	84	29.8	21	29.2	51	28.6
		Nutrition survey	52	18.4	13	18.1	32	18
		Other survey	107	37.9	17	23.6	75	42.1
		Denominator estimation	83	29.4	20	27.8	52	29.2
		Needs assessment	101	35.8	24	33.3	66	34.1
		Risk assessment	152	53.9	34	47.2	98	55.1
		Managing complex datasets	78	27.7	23	31.9	47	26.4
		R	34	12.1	12	16.7	18	10.1
		Stata	123	43.6	28	38.9	79	44.4
		Epi Info	205	72.7	31	43.1	149	83.7
		Excel	187	66.3	35	48.6	131	73.6
		Data visualisation	112	39.7	26	36.1	67	37.6
		Transmission trees	36	12.8	11	15.3	20	11.2
		Spatial analysis	71	25.2	19	26.4	38	21.4
c	Training in leadership and management							
		Evidence-based decision-making	159	56.4	35	48.6	108	60.7
		Mentoring	98	34.8	10	13.9	74	41.6
		Leadership	130	46.1	19	26.4	91	51.1
		Managing a team	119	42.2	20	27.8	85	47.8
		Peer teaching	93	33	9	12.5	70	39.3
		Team work	184	65.2	33	45.8	126	70.8
		Prioritisation	87	30.9	17	23.6	60	33.7
		Delegating responsibility	73	25.9	13	18.1	52	29.2
		Partner coordination	84	29.8	15	20.8	60	33.7
		Reflective practices	33	11.7	8	11.1	21	11.8
d	Training in social and communication skills							
		Basic scientific communication skills	244	86.5	48	66.7	166	93.3
		Practical field communication skills	149	52.8	24	33.3	107	60.1
		Media communication	136	48.2	24	33.3	94	52.8
		Cultural competency	72	25.5	22	30.6	42	23.6
		Participant consent	152	53.9	30	41.7	100	56.2
		Stress management	60	21.3	9	12.5	43	24.2
		Social media	32	11.3	5	6.9	22	12.4
		Interview techniques	144	51.1	31	43.1	95	53.4
		Ethics	169	59.9	40	55.6	111	62.4
		Relationship building	62	22	14	19.4	38	21.3
e	Training in emergency response training							
		Role of the epidemiologist during emergency response	169	59.9	33	45.8	113	63.5
		Epidemiology of public health disasters	166	58.9	32	44.4	114	64
		Humanitarian principles	67	23.8	18	25	42	23.6
		Principles of escalation/scaling a response	45	16	9	12.5	30	16.9
		Methods of data collection in an emergency	147	52.1	31	43.1	96	53.9
		Ethics during emergencies	82	29.1	18	25	53	29.8
		IMS—Incident Management System	73	25.9	14	19.4	46	25.8
		EOC role—Emergency Operations Centre	93	33	17	23.6	63	35.4
		IHR—International Health Regulations	121	42.9	15	20.8	87	48.9
		HeRAMS: Health Resources Availability Monitoring System	11	3.9	1	1.4	8	4.5
		EWARS: Early Warning, Alert and Response System (EWARS in a box)	79	28	12	16.7	58	32.6
		Personal safety (use of PPE—personal protective equipment)	130	46.1	26	36.1	88	49.4
		Border control (POE—point of entry)	46	16.3	8	11.1	30	16.9

Respondents highlighted the need for additional opportunities for knowledge consolidation and skill development in both outbreak and surveillance, specifying the need for teaching grounded within the political and social context (Box 1). Additional training was requested on responding to less common events such as environmental, chemical and radiological disasters (Box 1).

#### Box 1. Outbreak and surveillance training gaps reported by epidemiology emergency response survey respondents, 2019–2020

**Table:** 

Application of practical skills	*“I feel we were given enough knowledge but did not apply it in practice.”*
	*“I believe that I wanted to have more opportunities to apply in practice what I learned theoretically.”*
	*“While we acquire lots of knowledge, the practical competencies could be more developed by being in the field during the training program”*
Course relevance	*[training] “was mostly based around field epidemiology in stable, developing world settings. It's mostly related to the work of government departments.”*
	*“The knowledge and skills learnt were more geared toward outbreak response … Even then, looking back they were somewhat outdated and didn't necessarily reflect the political culture within which outbreaks occur.“ *
Knowledge	*[I wanted training] “related to chemical, radiological and explosive emergencies”*

### Data analysis and epidemiology methods

Fifteen emergency response training categories were listed in the survey (Table 3b). Reported training in specific methodology and data analysis skills needed during emergency response was limited. Training on estimation of population size during emergencies was reported by 29.4% (*n* = 83/282), which was similar for both FETP (29.2% *n* = 52/178) and non-FETP (27.8% *n* = 20/72) respondents. Thirty-six percent (*n* = 101/282) of respondents reported receiving training on how to conduct a needs assessment, and 53.9% (*n* = 152/282) on how to conduct a risk assessment. Training in survey development and implementation of specialised survey methods, such as nutrition and mortality surveys, were uncommonly reported (18.4% *n* = 52/282, and 29.8% *n* = 84/282, respectively), as were analytical techniques such as transmission trees (12.8% *n* = 36/282) and spatial analysis (25.2% *n* = 71/282) (Table 3b). Only 28% (*n* = 78/282) of respondents had received training in managing complex datasets.

The most common statistical and data management packages that respondents reported learning during their training was Epi Info™ (72.7% *n* = 205/282), followed by Microsoft Excel (66.3% *n* = 187/282) and Stata Statistical Software (43.6% *n* = 123/282) (Table 3b). A higher proportion of FETP graduates were trained in statistical packages compared to non-FETP graduates, with 83.7% (*n* = 149/178) of FETP trained respondents taught Epi Info™ compared to 43.1% (*n* = 31/72) non-FETP. Microsoft Excel training was reported by 73.6% (*n* = 131/178) of FETP trained respondents compared to 48.6% (*n* = 35/752) of non-FETP respondents.

Respondents highlighted additional training needs in specific software increasingly used in applied field epidemiology; specifically, training in R statistical software, population denominator estimation, spatial analysis, and geo-spatial mapping.

### Leadership and management

Ten leadership and management training categories were listed in the survey (Table 3c) Sixty-five percent (*n* = 184/282) of respondents reported having received training in teamwork, and 56.4% (*n* = 159/282) reported training in evidence-based decision-making. Responses to the remaining eight items in the leadership and management module of the survey indicated that these were missing from most training programs. Less than 50% of respondents reported having learnt leadership and management skills during their applied epidemiology training (Table 3c). For example, only 11.7% (*n* = 33/282) of respondents reported training in skill development related to reflective practices.

Although leadership training was not widely reported, FETP trained respondents reported a higher percentage of training in all leadership categories compared to non-FETP, especially in mentoring and peer teaching (Table 3c). Within each category in this section, males reported much higher leadership and management exposure, with the exception of peer teaching. Of note, during training 45.9% (*n* = 62/135) of male respondents reported having been taught mentoring, compared to 24.3% (*n* = 35/144) of female respondents, 61.5% (*n* = 83/135) of males reported training in leadership skills compared to 31.3% (*n* = 45/144) of females, and 34.1% (*n* = 46/135) of males reported training in delegation skills compared to 17.4% (*n* = 25/144) of females (Table 4).

**Table 4:** Reported training in leadership and management, comparison of epidemiology emergency response survey by gender, 2019–2020 (*n* = 282)

Topic	Female *n* = 144 (%)	Male *n* = 135 (%)
Evidence-based decision-making	77 (53.5%)	80 (59.3%)
Mentoring	35 (24.3%)	62 (45.9%)
Leadership	45 (31.3%)	83 (61.5%)
Managing a team	45 (31.3%)	72 (53.3%)
Peer teaching	51 (35.4%)	42 (31.1%)
Team work	89 (61.8%)	92 (68.1%)
Prioritisation	38 (26.4%)	47 (34.8%)
Delegating responsibility	25 (17.4%)	46 (34.1%)
Partner coordination	37 (25.7%)	45 (33.3%)

### Social and communication skills

Ten training categories were listed in the survey under social and communication skills (Table 3d). The majority (86.5% *n* = 244/282) of respondents reported training in basic scientific communication such as report writing and presentation preparation. Training in other communication techniques was less frequently reported; including training in practical communication in the field (52.8% *n* = 149/282), media communication (48.2% *n* = 136/282), cultural competence (25.5% *n* = 72/282), and social media communication (11.3% *n* = 32/282) (Table 3d). Approximately 20% of respondents reported training in stress management (*n* = 60/282) and relationship building (*n* = 62/282). Interview techniques, issues related to consent, and practical ethical approaches to field work were reported by just over half of the respondents (Table 3d). With the exception of cultural competence, non-FETP trained participants reported less training than FETPs to each category in this section (Table 3d).

Free text responses highlighted the need for more training in social and communication skills, (Box 2). Additionally, respondents indicated the need for further training in traditional media and social media. (Box 2). Respondents also stated that training with a focus on cultural, political, and contextual understanding was needed, as was training on qualitative research methods, stress management, and prioritisation (Box 2).

#### Box 2. Social and communication training gaps reported by epidemiology emergency response survey respondents, 2019–2020

**Table:** 

Communication	*“I think a really valuable skill that should be taught in epidemiology is how to influence others with the data story. This isn't about most fancy, complicated analysis, but about working out what's important in the data for decision-making”*
Social science and anthropological skills	*“My training as an anthropologist and social scientist significantly enhanced my skills as an epidemiologist. These are new concepts and under recognized and underused”*
	*“I will welcome more knowledge in behavioural change and anthropology during the training”*

### Emergency response

Fourteen emergency response training items were listed in the survey with half (*n* = 7) of them reported by less than 30% of respondents each (Table 3e). Non-FETP respondents reported less training than FETP graduates in each of these emergency response categories (Table 3e).

When respondents were asked whether they believed emergency response training should be a core component of applied epidemiology training, almost 75% (*n* = 211/282) replied yes, and 18% (*n* = 50/282) suggested it should be optional.

**Fig. 3:**
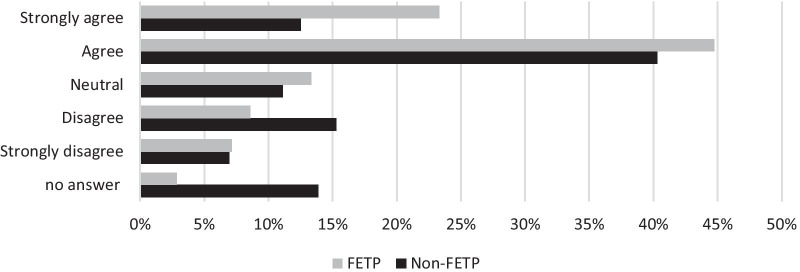
FETP and non-FETP respondents' perceptions of epidemiology training providing the knowledge required for emergency responses (*n* = 282)

**Fig. 4:**
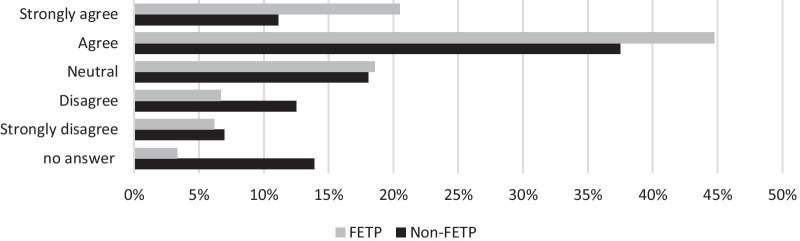
FETP and non-FETP respondents' perceptions of epidemiology training providing the skill required for emergency responses (*n* = 282)

Despite relatively low proportions of respondents having received formal training in the listed emergency response categories, 64% (*n* = 181/282) agreed or strongly agreed that their epidemiology training gave them the required knowledge to work as an epidemiologist during emergency response (Fig. 3). Additionally, 65% (*n* = 172/282) believed they had learnt the required skills for epidemiology emergency response (Fig. 4). FETP respondents were more likely to answer positively to these questions than non-FETP (Figs. 3 and 4).

Respondents requested additional training on the role of an epidemiologist during emergency response, personal and professional characteristics needed during a response, epidemiology methods in emergency response, humanitarian principles, as well as training on the emergency response structure (Box 3).

#### Box 3 Emergency response training gaps reported by epidemiology emergency response survey respondents, 2019–2020

**Table:** 

Emergency response	“*Being fit for the field, i.e. not everyone who has completed the program should be in the field*”
	“*Specific things like bioterrorism and natural disasters and hurricane response*”
	“*Critical skills to data analysis and collection during an emergency, rarely having a denominator, working with dirty data, working with MoH's [Ministry of Health] without statistical packages*”
	“*Did not learn enough about the different approaches and methods that are/should be used in an emergency setting vs a regular ongoing surveillance or outbreak response setting*”
	“*Thinking systems in the middle of an emergency*”
	“*A session on the realities of an emergency situation would be useful*”
	“*There was minimal relevant to emergency response or low resource settings*”
Response structure	“*How humanitarian responses are structured—what is happening around you in such a response and concrete feedback about where epi skills and information can serve those components*
	“*There were no emergency response topics covered in my epi training… no mention of IHR [International Health Regulations]*” *“*
Roles and responsibilities	*Role of Field Epidemiologist during Humanitarian crisis still confuses me”*
	“*Even at the advanced level and despite being "on the ground", does not have the capacity of those trained in the roles of the epidemiologist in emergency situations or health crises, in rapid interventions*”
	“*In a situation with an environmental disaster (e.g. air pollution), the epidemiologist has no preparation on his role in such an event. Other areas: Mass casualty, major water contamination, exposure to chemicals due to explosions, etc... responders are prepared, but the epidemiologist is not*”

## Discussion

Our survey on epidemiology training for emergency response identified core training needs of the applied epidemiology workforce. Our findings indicate that respondents believe their training in outbreak and surveillance, regardless of different training models, was sufficient. Respondents did, however, identify training gaps in the areas of social and communication skills, leadership and management, data analysis, epidemiology methods, and emergency response. Additionally, respondents identified that training methods needed to focus more on application of practical skills.

The COVID-19 pandemic has been another reminder of the importance of communication skills in connecting with the public during a public health emergency and the essential role of effective communication in the successful implementation of control measures [26–28]. Applied epidemiologists are often required to draw on a variety and diverse range of communication skills in order to effect change. These skills range from communicating risk to the public, communicating methods and findings to peers and designing messages that will support policy and decision-makers to implement an effective public health response. As the applied epidemiology definition by Thacker and Buffington [6] states, applied epidemiology is about “transforming findings to policy and action”, and as one of the respondents wrote, *“it is not just about collecting data but it is about influencing others with the story”*. Our survey identified gaps in applied epidemiology training in the areas of cultural competence, understanding the socio-political context, and anthropological principles. This training deficit was documented in a study of emergency response epidemiologists who responded to the 2014–2016 West Africa Ebola crisis [19], and a study looking to understand outbreak investigation training [29]. Despite the crucial need for these skills to function optimally as applied epidemiologists, survey respondents highlighted that these skills were not routinely taught in their applied epidemiology training.

Leadership and management skills are crucial for responding effectively and efficiently to public health events. One of the primary aims of FETPs is to train future public health leaders [8, 30, 31], with leadership and management listed as a core FETP competency. However, all training categories in the leadership and management section of our survey were uncommonly reported by respondents, with the exception of working in a team. Holding et al. [19] study post the West Africa Ebola crisis found a similar deficit in leadership training, as did Samet and Brownson [32] in their study of epidemiology graduates in the United States. A key characteristic of applied epidemiology is making evidence-based decisions, however only about half of respondents reported having received training on this.

While we did not identify any significant difference between respondents in regards to gender, level of education, or type of education, we identified significant difference in leadership and decision-making training between males and females. In this self-reported survey, this raises questions on whether leadership has the same meaning or interpretation across genders. The difference in response between genders may be linked to stereotypes and individual definitions around what constitutes leadership [33–35]. Traditionally, leadership has been linked to masculine traits, which then also devalue the feminine style of leadership [34, 35]. Johnson and Blair discuss how COVID-19 is shifting this understanding of leadership [34]. This concept of gender is important for leadership in emergencies and associated training needs further exploration through operational research.

When focusing on technical epidemiological skills, few respondents reported training in estimation of population density or specialised surveys such as mortality or nutrition surveys. Médecins Sans Frontières (MSF) have listed key competencies for epidemiologists in emergency response; including experience in practical applied research methods, as well as survey development and implementation (including mortality and nutritional surveys) [36]. Few respondents reported having received training in these specialised skills. Graduates of FETP programs who are trained to respond to public health events, without training in population estimation, population surveys, risk and needs assessment, would struggle to respond effectively to public health emergencies. Standardisation of FETP curriculum and core concepts regarding emergency response is urgently needed [37].

In addition, respondents to our survey highlighted the need to further understand the role of an epidemiologist during an emergency response, and the emergency response structure. Emergency response, whether it be a local or international public health emergency, should be a core competency of applied epidemiologists, who are often early responders. To be adequately equipped, this workforce needs more specialised skills in emergency response which appear to be neglected in the current models of applied epidemiology training. Continuous professional development activities should be considered for this workforce to ensure skills and knowledge is maintained, developed and responsive to changes in the field epidemiology emergency response landscape.

In our survey, there was an evident lack of training in practices that support the applied epidemiology workforce to remain strong and healthy and prevent burnout, such as stress management and reflective practices. Many countries struggle to retain field epidemiologists due to occupational burnout or career path limitations [7, 31, 38]. Ryu et al. [7] study on field epidemiology occupational stress, suggests that burnout is a very real concern given the frontline nature of outbreak and emergency response.

Although this survey focused on individuals’ training experience, our findings may be used to explore challenges to the broader workforce. We need to consider what capacities are required for the management and control of public health emergencies, not just the individual needs of each trainee [39]. Investment in a resilient applied epidemiology workforce means ensuring the workforce have the skills and knowledge needed to fulfil the various health security roles they will play now and in the future [5, 29, 32, 37].

## Study limitations

It is important to recognise some limitations with this study that may impact on our findings. We collected data through an opt-in self-administered online survey. The survey was not offered in any other format, therefore those without internet access were not able to participate, which means we may have excluded people working at the community level. We attempted to limit the effect of selection-biases introduced because of our approach to sampling by using multiple sources and methods to recruit participants. We offered the survey in two languages, French and English, as these are the common languages used during recent international emergency responses. We are cautious in generalising our findings to the applied epidemiology workforce, however, our sample size was large enough to identify common categories.

It was difficult for us to identify an accurate study target population sampling frame. The applied epidemiology workforce in most countries is informal and often poorly documented with the definition of ‘epidemiologist’ varying between individuals and organisations. The work title of an epidemiologist is largely through self-identification. Due to this limitation, we designed a cross-sectional survey distributed through professional networks. The study target population was defined as anyone who self-identified as having worked in an applied epidemiology role. However, because of this design we are unable to comment on how survey respondents differed from non-respondents.

As the timing of the survey was not during or immediately after the respondents’ training in epidemiology, recall bias may have affected the information obtained. Respondents also had varying years and types of experience and time since graduation. This gap between training and our survey may have been advantageous as respondents may have had time to reflect on what went well and what could be improved [40, 41].

In recent years, FETP training has expanded to three levels including a frontline and intermediate program, although few representatives from these programs accessed the survey. Future applied epidemiology workforce surveys should be targeted at graduates of these programs. Despite these limitations, our study offers insight into gaps in training for applied epidemiologists, which is supported by other operational research.

## Conclusion

Our survey identified that applied epidemiology training needs to evolve to provide capacity and skills to respond to dynamic and complex public health emergencies. There is a need to address the identified training gaps in leadership, communication and social skills, as well as emergency response. This will strengthen the applied epidemiology workforce, as well as the health systems they function within and the local, national, regional and global emergencies they respond to. Continuous professional development activities must also be available to support the current workforce adapt, as well as augment new graduates to be suitably skilled for the challenges ahead.

## Data Availability

All data relevant to this manuscript are included within the manuscript tables, figures and/or reference links.

## Abbreviations

AFRO: Regional Office for Africa
COVID-19: Coronavirus Disease 2019
EMRO: Regional Office for the Eastern Mediterranean
EOC: Emergency Operations Centre
ESCAIDE: European Scientific Conference on Applied Infectious Disease Epidemiology
EURO: Regional Office for Europe
EWARS: Early Warning, Alert, and Response System
FETP: Field Epidemiology Training Program
HeRAMS: Health Resources Availability Monitoring System
IHR: International Health Regulations
IMS: Incident management systems
MPH: Master of Public Health
MSF: Médecins Sans Frontières
PAHO: The Pan American Health Organization
POE: Point of Entry
PPE: Personal protective equipment
REDCap: Research Electronic Data Capture
SEARO: South-East Asia Regional Office
TEPHINET: Training Programs in Epidemiology and Public Health Interventions Network
TEPHIConnect: TEPHINET alumni network
WHO: World Health Organization
WPRO: Western Pacific Region Office
